# Ageing, health system implications, and a changing epidemic: organizational transitions and stigma dynamics in Philippine HIV care

**DOI:** 10.3389/fpubh.2026.1857117

**Published:** 2026-06-25

**Authors:** Steven Stumph, Paul Nash, Louie M. A. Gangcuangco

**Affiliations:** 1 Leonard Davis School of Gerontology, University of Southern California, Los Angeles, CA, United States; 2 Waikiki Health, Honolulu, HI, United States

**Keywords:** ageing with HIV, ageism and age-based discrimination, health literacy (HL), health system—organization and administration, HIV—human immunodeficiency virus, Philippines, sexuality and related issues, stigma and discrimination

## Abstract

**Background:**

The global HIV epidemic is undergoing a demographic transition as expanded access to antiretroviral therapy (ART) has increased life expectancy among people living with HIV (PLWH). As a result, the population of older adults living with HIV (OPLWH) is rapidly growing, introducing new clinical, psychosocial, and public health challenges. In the Philippines, one of the fastest-growing HIV epidemics in the Asia-Pacific region, this shift is occurring alongside rising incidence, creating a dual burden of ongoing transmission and emerging survivorship needs.

**Methods:**

This study employed a structured narrative review and policy-oriented conceptual synthesis of multidisciplinary literature published between 2010 and 2025. An ecological framework guided analysis across individual, interpersonal, community, and structural levels, with emphasis on accelerated and accentuated ageing, multimorbidity, stigma, and health literacy (HL). The Optimizing Health Literacy and Access (OPHELIA) framework was applied to translate findings into equity-centered public health strategies.

**Results:**

OPLWH in the Philippines face compounded vulnerabilities driven by biological ageing, multimorbidity, and intersecting forms of stigma, including ageism and HIV-related discrimination. Structural barriers such as fragmented health systems, workforce limitations, geographic inequities, and limited HL capacity, constrain sustained engagement in care. Diagnostic invisibility and late presentation among older adults persist, while inadequate age-disaggregated surveillance limits recognition of ageing-related burden. Psychosocial factors, including loneliness, internalized stigma, and cultural norms surrounding sexuality and ageing, further influence adherence, care retention, and quality of life.

**Conclusion:**

The findings underscore the need for integrated, age-responsive HIV care models that incorporate geriatric principles, mental health services, and culturally grounded stigma reduction. Expanding the OPHELIA framework demonstrates how HL-informed, community co-designed interventions can strengthen care continuity and survivorship outcomes. Policy priorities include workforce training in HIV and gerontology, improved age-disaggregated surveillance, HL interventions, and climate-resilient service delivery. Addressing ageing with HIV in the Philippines requires coordinated, system-level reform to ensure that increased longevity is accompanied by equitable, sustained health outcomes.

## Introduction

Human immunodeficiency virus (HIV) continues to remain a substantial global health burden despite major advances in prevention, testing, and treatment. Since the beginning of the epidemic more than four decades ago, HIV has claimed an estimated 40 million lives worldwide (1). Although global incidence and mortality have declined compared with peak epidemic periods, approximately 1.6 million new infections and more than 700,000 HIV-related deaths continue to occur annually, with an estimated 38–40 million people currently living with HIV (PLWH) worldwide (2). Sustained expansion of antiretroviral therapy (ART) has transformed HIV from a rapidly fatal disease into a chronic and increasingly manageable condition for many individuals, substantially improving survival and shifting the global HIV response toward long-term disease management and continuity of care (3, 4). As treatment access improves, health systems must increasingly address not only viral suppression, but also the broader biological, psychosocial, and structural dimensions of ageing with HIV.

One of the most significant consequences of ART success has been the demographic transformation of the HIV epidemic. The global population of older adults living with HIV (OPLWH), commonly defined as adults aged 50 years and older, continues to increase rapidly (5). Estimates suggest that between five and eight million PLWH worldwide are now aged 50 years or older, with the proportion expected to rise over the coming decades (5, 6). As survivorship improves, HIV intersects with age-related multimorbidity, frailty, neurocognitive decline, mental health challenges, and social isolation (7, 8). OPLWH may experience accelerated and accentuated ageing processes associated with chronic immune activation, persistent inflammation, and long-term treatment exposure, contributing to earlier onset and increased prevalence of age-associated comorbidities compared with HIV-negative populations (9, 10). Simultaneously, psychosocial factors including stigma, loneliness, depression, and structural inequities may further shape long-term survivorship trajectories (11, 12). Consequently, ageing with HIV represents not solely a biomedical phenomenon, but a multidimensional public health challenge requiring integrated and equity-centered responses.

Within Asia, the HIV epidemic remains concentrated among key populations, including men who have sex with men (MSM), people who inject drugs, sex workers, transgender and gender-diverse individuals, and other socially marginalized groups (13, 14). However, important regional differences exist in epidemic trajectory and health system preparedness. Countries such as Thailand have demonstrated sustained reductions in HIV incidence through long-term investments in prevention, testing, and community-based interventions (15). In contrast, the Philippines has experienced one of the fastest-growing HIV epidemics in the Asia-Pacific region over the past decade (16, 17). While the epidemic in the Philippines remains concentrated among younger MSM and other key populations, increasing ART access is simultaneously expanding the population of long-term survivors. The country therefore faces a dual epidemiologic and public health challenge: responding to ongoing transmission while preparing for the complex survivorship needs of an ageing HIV population.

The Philippine context presents several distinct structural and sociocultural challenges relevant to ageing with HIV. The national health system remains fragmented across decentralized administrative structures and geographically dispersed islands, creating barriers to continuity of care, specialist referral systems, and sustained ART engagement (18). Workforce shortages, uneven digital infrastructure, and geographic disparities further complicate access to long-term HIV and geriatric services (19). At the same time, HIV-related stigma remains an important determinant of testing behaviors, disclosure, treatment engagement, and psychosocial well-being within the Philippines (13, 20). For older adults living with HIV (OPLWH), these challenges may be compounded by ageism, social isolation, and assumptions that HIV primarily affects younger populations (21). Consequently, ageing with HIV must be understood not only through biomedical frameworks, but also through the social and structural contexts that shape long-term survivorship.

Health literacy (HL) further shapes survivorship outcomes within the Philippine HIV response. Effective long-term HIV management requires individuals to navigate complex treatment regimens, multimorbidity care pathways, insurance systems, digital health platforms, and evolving prevention concepts such as Undetectable = Untransmittable (U=U). Older adults face additional barriers related to sensory decline, neurocognitive vulnerability, limited digital familiarity, and fragmented service systems (22, 23). Consequently, ageing with HIV must be understood not only through clinical and epidemiologic frameworks, but also through broader ecological systems involving HL, digital inclusion, stigma, social support, and structural accessibility.

The growing convergence of ageing, HIV survivorship, multimorbidity, and health system strain highlights the need for integrated and age-responsive public health frameworks. Historically, HIV services and geriatric care systems have operated in parallel rather than through coordinated multidisciplinary models (24). In low- and middle-income settings such as the Philippines, where resources remain constrained and age-disaggregated HIV data remain limited, these challenges may be amplified. Existing evidence regarding accelerated ageing, frailty, neurocognitive decline, and multimorbidity among PLWH derives largely from high-income-country cohorts, raising important questions regarding transferability to the Philippine context, where differences in health system capacity, coinfection burden, socioeconomic vulnerability, and continuity of care may influence ageing trajectories differently (25, 26). The limited availability of longitudinal ageing-specific HIV data in the Philippines further underscores the need for contextually grounded public health analysis.

This study therefore examines ageing with HIV in the Philippines through an ecological and public health systems lens. Drawing on multidisciplinary literature across epidemiology, ageing biology, psychosocial survivorship, stigma, HL, and health systems research, the manuscript synthesizes the emerging challenges associated with OPLWH within the Philippine context. Particular attention is given to accelerated and accentuated ageing, multimorbidity, mental health, intersectional stigma, digital inclusion, and structural fragmentation within HIV care systems. To translate these findings into actionable implementation pathways, the study applies and expands the Optimizing Health Literacy and Access (OPHELIA) framework as an organizing model for age-responsive and equity-centered HIV survivorship planning. Through this integrated approach, the manuscript aims to contribute to emerging public health discussions surrounding ageing with HIV and to strengthen preparedness for the evolving demographic realities of the Philippine HIV epidemic.

## Methodology

This study employed a structured narrative review and policy-oriented conceptual synthesis to examine ageing with HIV in the Philippines, with particular emphasis on stigma, HL, mental health, and health system preparedness. A narrative approach was selected because the evidence base spans heterogeneous study designs, including epidemiologic trend analyses, qualitative research, clinical ageing studies, implementation science, and policy documents. The aim was to synthesize multidisciplinary evidence and translate it into a contextually grounded public health framework relevant to the Philippine setting and the emerging population of OPLWH, rather than generate pooled quantitative estimates.

The review was conducted between November 2025 and March 2026 and included literature published from January 2010 through December 2025 to capture contemporary epidemiologic developments, advances in ageing science, and evolving health system reforms. Searches were conducted in PubMed/MEDLINE, Scopus, Web of Science, CINAHL, and Google Scholar. Grey literature was identified through targeted searches of institutional repositories, including those of the Philippine Department of Health, UNAIDS, the World Health Organization, and the World Bank. Reference lists of included articles were also manually screened for additional relevant publications.

Titles and abstracts were screened for relevance to ageing, stigma, survivorship, mental health, HL, and structural determinants of HIV care. Potentially eligible studies then underwent full-text review to confirm relevance and eligibility. Screening and thematic categorization were conducted iteratively using an ecological analytic framework across individual, interpersonal, community, and structural levels. Given the conceptual breadth of the review and heterogeneity of the evidence, formal risk-of-bias scoring was not applied. Instead, methodological rigor, contextual relevance, analytic clarity, and applicability to low- and middle-income settings informed interpretive synthesis.

Search strategies combined HIV- and Philippines-related terms with concepts addressing ageing, survivorship, stigma, multimorbidity, mental health, and health systems. Keywords included variations of “HIV,” “Philippines,” and “Filipino,” paired with terms such as “aging,” “older adults,” “geriatric,” “frailty,” “multimorbidity,” “neurocognitive,” “accelerated aging,” and “immunosenescence.” Additional terms captured psychosocial and structural dimensions, including “stigma,” “ageism,” “loneliness,” “mental health,” “health literacy,” “digital literacy,” “service delivery,” “integrated care,” “Universal Health Care,” and “digital health.” Search syntax was adapted to database-specific indexing conventions.

Studies were eligible for inclusion if they were published in English between 2010 and 2025 and examined HIV epidemiology, prevention, survivorship, stigma, ageing, mental health, or health system adaptation in the Philippines. Given the limited age-disaggregated literature specific to older adults in the Philippine context, global and regional studies addressing accelerated or accentuated ageing among PLWH were also included when they offered conceptual or clinical insights applicable to low- and middle-income settings. Quantitative, qualitative, mixed-methods, policy, and implementation studies were included if they contributed to understanding multilevel determinants of HIV risk, care engagement, survivorship, or ageing-related vulnerability. Grey literature was included when it came from recognized public health institutions and contributed substantively to epidemiologic or policy context.

Studies focusing exclusively on pediatric populations or case reports without broader analytic relevance were excluded, as were articles from high-income settings without clear conceptual applicability to resource-constrained contexts. Nevertheless, some mechanistic discussions of accelerated ageing, multimorbidity, frailty, and neurocognitive decline necessarily drew on high-income-country cohorts because longitudinal ageing data specific to the Philippines remain limited. These findings should therefore be interpreted cautiously, and future Philippine-specific validation studies are needed. Evidence was organized into four thematic domains: biological and clinical dimensions of ageing with HIV, psychological and mental health dimensions, social and structural determinants of survivorship, and translational and health system response pathways.

An ecological analytic lens guided interpretation of findings. Drawing on Bronfenbrenner’s (27) ecological systems theory and the modified social ecological model for HIV risk and care engagement (28–30), determinants were synthesized across individual, interpersonal, community, and structural levels. Within this framework, accelerated and accentuated ageing were used to interpret biological and psychosocial vulnerability among OPLWH.

**Figure 1:**
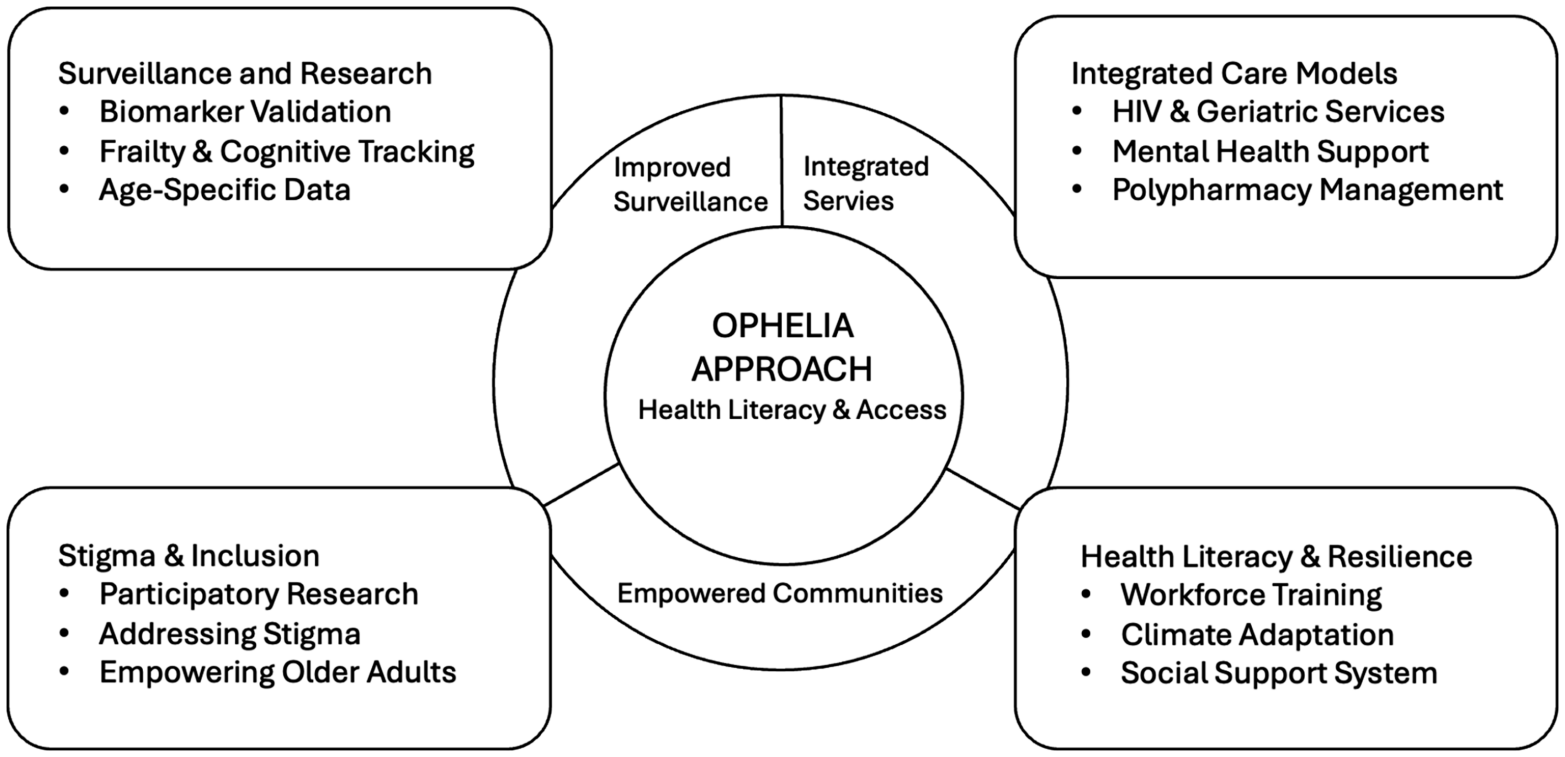
Integrated OPHELIA Approach. The OPHELIA model with an expanded framework on health literacy and access for HIV and ageing in the Philippines.

To translate the synthesized evidence into implementation-relevant strategy, the OPHELIA framework was used as a secondary organizing model. The original OPHELIA framework emphasizes situational assessment, co-design of interventions, and iterative implementation responsive to population HL strengths and limitations. In this manuscript, the framework is expanded to incorporate age-responsive HIV survivorship, multimorbidity management, digital inclusion, and climate-resilient continuity planning within the Philippine context. The expanded OPHELIA approach functions as a translational implementation model linking ecological determinants with actionable public health and health system interventions (see Figure 1).

### Structured narrative review process and methodological transparency

An underrecognized challenge within the ageing HIV epidemic is the persistent diagnostic invisibility experienced by older adults. Although HIV prevention and testing initiatives in the Philippines have appropriately focused on younger key populations, particularly MSM, this emphasis may inadvertently reinforce the perception that HIV is primarily a disease of youth (31). As a result, older adults may be overlooked in prevention messaging, risk communication, and routine screening despite ongoing sexual activity and potential exposure risk, contributing to delayed diagnosis and advanced disease presentation.

Several factors may contribute to later-stage HIV diagnoses among adults aged 50 years and older. Older adults may underestimate personal HIV risk due to assumptions surrounding ageing and sexuality, while providers may overlook HIV when evaluating symptoms such as fatigue, weight loss, recurrent infections, or neurocognitive changes. Consequently, HIV-related symptoms may be attributed to normative ageing or chronic disease rather than prompting testing or referral. Broader societal assumptions that older adults are sexually inactive or outside traditional HIV “risk groups” may further discourage discussions of sexual health and limit screening opportunities (32). Within the Philippine context, where discussions of sexuality are often shaped by conservative cultural and religious norms, older women, widowed individuals, heterosexual men, and those re-entering intimate relationships later in life may remain underserved by prevention initiatives despite ongoing vulnerability.

Delayed diagnosis has important implications for both survivorship and health system preparedness. Prolonged untreated infection may contribute to cumulative immune activation, increased multimorbidity burden, and greater risk of ageing-related complications, while late presentation may complicate management through concurrent chronic illness, polypharmacy, neurocognitive vulnerability, and delayed ART initiation (33). Diagnosis later in life may also intensify internalized stigma and shame where HIV remains associated with moralized narratives surrounding sexuality and personal behavior.

These challenges underscore the need for age-disaggregated HIV surveillance in the Philippines. Current surveillance systems often aggregate older adults into broad age categories, limiting identification of trends involving viral suppression, retention in care, multimorbidity, frailty, neurocognitive decline, and survivorship outcomes among OPLWH (34). More granular age-stratified surveillance may strengthen workforce planning, chronic disease forecasting, and allocation of survivorship resources, particularly if geriatric indicators such as frailty, cognitive screening outcomes, and multimorbidity burden are incorporated into routine monitoring.

Surveillance reform alone, however, is insufficient without corresponding improvements in provider awareness and clinical preparedness. Clinicians working within primary care, HIV services, and geriatric settings require training in age-inclusive sexual history taking, stigma-sensitive communication, recognition of atypical HIV presentations, and the unique survivorship needs of older adults (35). Within the expanded OPHELIA framework, surveillance reform and provider preparedness are complementary components of an age-responsive HIV response. Together, they may reduce diagnostic invisibility, improve earlier engagement in care, and strengthen long-term survivorship outcomes among OPLWH in the Philippines.

### Methodological limitations

Several limitations are inherent in this review design. As a structured narrative synthesis, the study does not provide pooled quantitative estimates and may be subject to selection bias. Although multiple databases and grey literature sources were searched, unpublished or non-English studies may not have been captured. Age-disaggregated data specific to OPLWH in the Philippines remain limited, constraining precise quantification of national ageing burden. Furthermore, much of the mechanistic evidence regarding accelerated biological ageing derives from international cohorts, and extrapolation to Filipino populations requires future empirical validation. Despite these limitations, the integrative approach allows for a multidimensional assessment of epidemiologic, biological, psychosocial, and structural determinants relevant to ageing with HIV in the Philippine context.

## HIV increase in the Philippines

The contemporary Philippines landscape reveals the structural and programmatic pressures that shape implementation of age-responsive strategies. Although survivorship is increasing and the population of OPLWH is expanding, the national response remains constrained by rising incidence, persistent care continuum attrition, uneven ART coverage, and reliance on mixed domestic and donor financing.

Recent surveillance data emphasizes the trajectory of the HIV epidemic in the Philippines. Between 2010 and 2017, HIV positivity rose from 16,000 to 68,000 cases, mainly among MSM, while testing and ART adherence declined from 61 to 28% during the same period (36). While domestic financing for HIV increased in recent years under Republic Act 11166 (37), the national response continues to rely substantially on external donor support for prevention commodities, laboratory systems, and technical assistance (38, 39). The trends in the Philippines show the urgency of strengthening financing, prevention, testing, and treatment strategies nationwide.

Attrition along the HIV care continuum remains a structural weakness in the Philippine response. Only two-thirds of individuals initiate ART usage within 3 months of diagnosis, and fewer than half achieve documental viral suppression (38). The gap reflects breakdowns in linkage, retention, medication continuity, and adherence support. Geographic distance to one of the 300 treatment hubs and primary HIV care treatment facilities nationwide creates recurring barriers for individuals requiring laboratory monitoring and prescription refills (34, 40). Patient satisfaction strongly predicts retention, including facility cleanliness, confidentiality, provider empathy, trust-building communication, and privacy-sensitive clinic locations influence whether patients remain engaged in care (40, 41).

Diversified testing strategies aim to address early diagnosis and linkage gaps, emphasizing accessible entry points into care. HIV self-testing (HIVST), recommended by the World Health Organization (WHO), expands confidential access for individuals concerned about stigma, provider mistrust, or scheduling barriers (42). In the Philippines, HIVST has successfully reached first-time testers and demonstrated cost-effectiveness among cisgender MSM, while older adults often prefer it due to privacy and convenience (31, 43, 44). However, linkage following a reactive self-test remains inconsistent, and sustained engagement in ART or pre-exposure prophylaxis (PrEP) after initial uptake is frequently low (4, 45). HIVST should be embedded within coordinated, literacy-sensitive systems that ensure immediate telehealth linkage, peer navigation, and facility-based confirmation pathways. Positioning HIVST as a complementary gateway supports continuity across the prevention to treatment spectrum.

Structural stigma continues to undermine progress across the care continuum and directly disrupt equity-centered design principles. HIV related discrimination in the Philippines, often rooted in moral judgments, religious beliefs, and stereotypes about sexuality, contributes to delayed testing, nondisclosure, and treatment interruption (13, 20, 46). Criminalization further compounds vulnerability (47). Sex workers and people who use drugs face legal risks that discourage engagement with formal health services, and methamphetamine (“*shabu*”) use within some sexual networks intensifies stigma where drug use intersects with sexual minority identity (36, 48, 49). Structural stigma represents a system level literacy and access barrier requiring co-designed, rights-based responses, which are depicted on mainstream media (50, 51). Harm reduction expansion, legal reform, and protective policies are not separate from clinical outcomes, but are foundational to reducing attrition and strengthening equitable engagement.

## Applying global HIV lessons to the Philippines

The lessons learned from other countries that have faced older HIV epidemics are applied to the Philippines where ageing with HIV is a newer and emerging challenge. The epidemiologic trajectory in the Philippines is characterized by rapid increases in new HIV infections, concentrated epidemics among key populations, and challenges in testing and linkage to sustained ART (52); projections suggest substantial growth in the population living with HIV through 2030 unless prevention and treatment gaps are closed (53). These national trends imply that the Philippines will increasingly confront the issues of ageing with HIV—albeit within local demographic, health-system, and socio-cultural contexts.

Insights from the U.S. and other high-income country research are valuable but require careful contextualization. First, the biological mechanisms (e.g., immunosenescence, epigenetic acceleration) appear broadly generalizable; molecular markers of ageing and immune activation have been observed across settings (25) and thus provide mechanistic targets for surveillance and intervention in the Philippines. Second, interventions shown to reduce cardiometabolic risk, improve physical function, or mitigate loneliness (e.g., structured exercise, social support groups, integrated primary–HIV care pathways) provide adaptable templates. However, differences in health-system capacity, ART coverage, prevalence of coinfections (e.g., TB, hepatitis), social stigma, and resource constraints necessitate locally tailored strategies.

For the Philippines, priority research and policy directions include epidemiologic surveillance that disaggregates age cohorts to identify early signals of accelerated/accentuated ageing (frailty indices, multimorbidity patterns, functional status) among PLWH and distinguishes lifetime survivors from those infected at older ages. Further, biomarker studies to validate epigenetic clocks, telomere measures, and inflammation panels in Filipino PLWH; such work will establish whether magnitude and timing of biological ageing observed in U.S. cohorts apply locally and will inform risk stratification. Implementation research is also imperative to test scalable interventions that integrate geriatric assessment into HIV clinics, adapt psychosocial interventions to local cultural norms (family caregiving patterns, community organizations), and mitigate stigma that impedes care engagement. Health-policy needs to reflect the research and be aligned for longitudinal care financing, workforce training (gerontology and HIV competencies), and cross-sectoral programs addressing social isolation (e.g., community centers, peer navigation). Finally, collaborations between Philippine investigators, local community stakeholders, and established research consortia in the U.S. can accelerate capacity building, transfer lessons on integrated care models, and co-develop culturally appropriate interventions. Emphasis should be placed on participatory research that centers the lived experiences of Filipino PLWH, leverages existing community networks, and evaluates outcomes meaningful to patients and families. Such an agenda will better position the Philippines to anticipate and reduce the burden of ageing with HIV in the coming decades.

## Biological and clinical dimensions in the Philippines

The global population of OPLWH continues to expand at an unprecedented rate, reshaping the demographic profile of the epidemic. In 2021, approximately 3.6 million individuals aged 50 years and older were living with HIV, representing 21% of all PLWH, compared to 1.5 million in 2016 (6). Despite the demographic shift, health systems have not consistently implemented differentiated service delivery (DSD) models tailored to older adults (6). OPLWH often experience high rates of comorbidities, alongside complex social and mental health needs that require age-responsive care models. DSD provides a framework for adapting HIV services to patient needs by integrating care, developing and validating screening tools and treatment algorithms for common comorbidities in high-burden settings, and scaling country-level innovations as services evolve (6).

Biological ageing further complicates the clinical management of HIV in later life. Ageing results from declining homeostatic maintenance and cellular repair mechanisms, disrupting multiple physiological systems, including the immune system. Chronic HIV infection has been associated with persistent immune activation, elevated inflammatory markers, and earlier onset of several age-associated comorbidities (5, 9). Emerging evidence suggests that HIV may contribute to biological ageing processes through mechanisms involving chronic inflammation and immune dysregulation, although the magnitude and timing of these effects remain areas of ongoing investigation. Long-term exposure to ART may influence physiological ageing processes through complex interactions involving chronic disease management, treatment duration, and cumulative health exposures. However, the extent to which ART independently contributes to ageing-related changes remains incompletely understood. Accurate evaluation of ageing with HIV requires attention to ethnicity, sex, lifestyle factors, and coinfections.

In addition to biological vulnerability, OPLWH face intersecting forms of social marginalization, including ageism. The WHO defines ageism as stereotypes, prejudice, and discrimination directed at individuals because of their age (54). Institutional ageism operates through laws, policies, social norms, and organizational practices that disadvantage people based on age, while self-ageism among people with HIV can manifest as diminished career expectations, survivor guilt, social isolation, loneliness, and internalized stigma shaped by sex and gender differences (24). Among PLWH, ageism can drive self-protective withdrawal from social relationships and sexuality, contributing to frailty, depression, loneliness, and reduced engagement in health care (54). These age-related barriers may contribute to delayed diagnosis and are discussed further in the section on diagnostic invisibility and provider preparedness. The HIV Outcomes EU initiative therefore calls for a comprehensive approach to ageing with HIV that promotes well-being, prevents comorbidities, and eliminates stigma and discrimination (54).

### Accelerated and accentuated ageing

The widespread availability of ART has transformed HIV from a fatal disease into a chronic condition for many people worldwide, contributing to a growing population of older adults living with HIV (OPLWH). Consequently, clinicians and researchers increasingly confront the challenges of accelerated and accentuated ageing. Accelerated ageing refers to an earlier onset of biological ageing processes following HIV infection, whereas accentuated ageing describes a greater prevalence or severity of age-associated conditions among PLWH compared with HIV-negative peers of the same chronological age. Together, these processes describe a pattern in which age-related morbidities appear to occur earlier and more frequently among PLWH (10).

As survivorship improves, the Philippine HIV epidemic is increasingly shaped by the long-term health needs of OPLWH. Sustained ART access, chronic disease management, cognitive screening, and mental health services are becoming essential components of care. At the same time, geographic fragmentation across the country’s 7,641 islands and decentralized governance structures complicate continuity of care (19). These demographic shifts highlight the need to understand ageing with HIV within the broader context of multimorbidity, survivorship, and health system capacity.

Addressing accelerated and accentuated ageing requires a multimodal approach. Earlier diagnosis and sustained viral suppression remain foundational. Longitudinal epigenetic studies suggest that untreated HIV infection is associated with accelerated biological ageing signatures, while sustained viral suppression may partially attenuate some methylation-based markers of ageing, although evidence regarding reversibility remains evolving (26). Comprehensive management of comorbidities, including cardiometabolic risk reduction, cancer screening, bone health assessment, medication review, and integration of geriatric principles such as frailty and cognitive screening, is also essential. Behavioral and psychosocial interventions—including structured exercise, smoking cessation, nutrition, cognitive stimulation, and programs that reduce loneliness and strengthen social networks—may provide additional benefits by improving functional status and attenuating pro-inflammatory states (55). Integrated care models and community-engaged interventions that address stigma and broader social determinants of health will be critical for achieving equitable outcomes.

The combined effects of accelerated biological ageing and adverse psychosocial conditions contribute to several interrelated challenges, including multimorbidity, neurocognitive decline, frailty, and HIV disease progression. Earlier onset of age-related chronic diseases increases the burden of multimorbidity and polypharmacy (56), complicating ART management and increasing the risk of drug–drug interactions.

### Contextual limitations of accelerated ageing research in low- and middle-income settings

Although substantial evidence supports the concepts of accelerated and accentuated ageing among PLWH, much of the existing biomarker, frailty, neurocognitive, and multimorbidity literature derives from cohorts in high-income settings, particularly the United States and Western Europe (25, 26). Consequently, caution is warranted when extrapolating these findings directly to the Philippine context and to other low- and middle-income settings where epidemiologic patterns, health systems, socioeconomic conditions, and continuity of care may differ substantially.

Several structural and contextual differences influence how ageing with HIV manifests in the Philippines. Health system capacity remains more fragmented than in many high-income settings, particularly across geographically isolated regions where continuity of HIV and chronic disease management may be inconsistent (18). Differences in specialist availability, referral systems, diagnostic infrastructure, and long-term survivorship services may influence the timing of diagnosis, treatment initiation, and management of multimorbidity among OPLWH. Workforce shortages and migration of healthcare professionals abroad may further constrain integrated HIV-geriatric care capacity (19).

Variability in ART continuity and treatment access may also shape ageing trajectories differently. While ART coverage has improved substantially in the Philippines, interruptions in linkage to care, geographic barriers to treatment hubs, medication continuity challenges, and delayed diagnosis remain ongoing concerns (38). In contrast, many biomarker and ageing studies from high-income countries are derived from cohorts with earlier ART initiation, longer periods of sustained viral suppression, and more stable continuity of care. Differences in cumulative untreated viremia and treatment interruption may therefore influence inflammatory burden, immune activation, and long-term survivorship outcomes in ways that are not yet fully understood within the Philippine context.

The burden and composition of multimorbidity may additionally differ across settings. In high-income countries, ageing PLWH commonly experience cardiovascular disease, metabolic disorders, osteoporosis, and neurocognitive decline associated with prolonged survivorship and ageing populations (56). However, in low- and middle-income settings, these conditions may coexist alongside infectious comorbidities, nutritional vulnerabilities, environmental stressors, and differing patterns of healthcare access (57). Coinfection prevalence, including tuberculosis, hepatitis infections, and recurrent communicable disease exposure, may further alter inflammatory and ageing trajectories among PLWH in the Philippines compared with cohorts from high-income settings.

Broader social determinants of health may also influence biological ageing processes and survivorship outcomes. Poverty, unstable housing, fragmented insurance access, stigma, social exclusion, disaster vulnerability, and uneven healthcare accessibility may contribute to chronic psychosocial stress and delayed engagement in care (58, 59). The structural pressures interact with biological ageing pathways in ways not fully captured within existing HIV ageing literature derived from resource-rich environments. Furthermore, experiences of stigma, family caregiving expectations, migration, and religious or cultural norms surrounding sexuality may uniquely shape psychosocial survivorship trajectories within the Philippines.

Diagnostic infrastructure limitations further complicate direct translation of biomarker findings. Advanced epigenetic ageing measures, inflammatory marker panels, neurocognitive testing platforms, and longitudinal frailty assessments commonly utilized in high-income cohort studies may not yet be routinely available or standardized within many Philippine healthcare settings. Consequently, the magnitude, timing, and clinical implications of accelerated ageing among Filipino PLWH remain incompletely characterized.

These contextual limitations emphasize the urgent need for Philippine-specific longitudinal and biomarker research examining ageing with HIV. Future studies should prioritize age-disaggregated surveillance, multimorbidity profiling, frailty assessment, neurocognitive trajectories, inflammatory biomarkers, and epigenetic ageing measures among Filipino PLWH. Longitudinal cohort development may allow more accurate characterization of survivorship patterns and strengthen understanding of how biological ageing interacts with local structural, psychosocial, and health system conditions. Such research may additionally inform development of culturally grounded and resource-responsive HIV-geriatric care models tailored to the Philippine setting.

Collaborative partnerships between Philippine investigators, HIV treatment hubs, geriatric specialists, community organizations, and international ageing research networks may provide important opportunities for capacity building and translational research development. Establishing locally grounded evidence regarding accelerated and accentuated ageing among Filipino PLWH will be critical for informing surveillance systems, workforce planning, multimorbidity management, and future survivorship-oriented public health policy.

### Multimorbidity

Ageing with HIV in the Philippines occurs alongside a rising burden of neurodegenerative disease. Approximately 10.6% of Filipinos aged 60 and older live with dementia (19). OPLWH face elevated risks of cognitive decline and HIV associated dementia (60). HIV establishes latency in the central nervous system and infects glial cells and astrocytes (61), contributing to dysfunction in various neurocognitive domains (62). Approximately 75% of reviewed studies report evidence of accelerated neurocognitive ageing among PLWH, although findings and exact rates of neurocognitive disorders remain mixed (60).

The patterns suggest that survivorship among OPLWH will involve multimorbidity, including cardiovascular disease, metabolic disorders, depression, and cognitive impairment (7, 11). Yet national projections estimating the future multimorbidity burden among ageing PLWH in the Philippines are limited. Explicit modeling of comorbidity clustering is needed to anticipate service demand.

Survivorship care must account for varying HL strengths and limitations related to cognitive decline. Declining executive function affect medication adherence, appointment keeping, and comprehension of evolving treatment regimens (23). Health systems must therefore incorporate routine neurocognitive and mental health screening into HIV services while adapting communication strategies to ensure comprehension and sustained engagement.

### Neurocognitive decline, frailty, and HIV disease progression

Neurocognitive vulnerability, frailty, and multimorbidity represent increasingly important dimensions of long-term HIV survivorship among OPLWH. Chronic immune activation, persistent inflammation, and psychosocial stressors have been associated with ageing-related functional decline among PLWH, although the precise mechanisms and magnitude of these relationships remain an active area of investigation (10). Emerging evidence suggests that psychosocial stressors may plausibly interact with inflammatory and neuroendocrine pathways, although much of the current evidence remains associative rather than definitively causal, particularly in low- and middle-income settings.

Frailty, falls, and cognitive impairment is observed among PLWH at younger chronological ages than historically expected within general population ageing models (63). HIV-associated neurocognitive vulnerability involves interactions between chronic immune activation, long-term treatment exposure, multimorbidity, vascular risk factors, and broader psychosocial determinants (60). However, most mechanistic and biomarker evidence regarding accelerated neurocognitive ageing derives from cohorts in high-income settings, and the extent to which these findings fully generalize to the Philippine context remains incompletely understood.

Although mechanistic understanding of inflammaging and immunosenescence continues to expand, effective and scalable interventions capable of safely modulating the suvivorship processes among PLWH remain limited (64). Consequently, future research should prioritize behavioral, pharmacologic, psychosocial, and systems-level interventions that reduce both biological and psychosocial ageing burdens among OPLWH. Greater emphasis on longitudinal survivorship monitoring, frailty assessment, neurocognitive screening, and integrated HIV-geriatric care models strengthens preparedness for the growing population of ageing PLWH in the Philippines.

## Psychological and mental health dimensions

Ageing with HIV is not solely a biological process but also the cumulation of social exposures across the life course. For older Filipinos ageing with HIV, the health system fragmentation increases cognitive and logistical burdens, further compounded by intense stigma, limited ART access, and high mortality visibility among peers in earlier epidemic phases (60, 65, 66). For those diagnoses at older ages, survivor’s guilt, anticipatory anxiety, and stigma are influenced by age-based assumptions about morality and sexual behavior (67–69), encouraging survivorship programming to incorporate narrative-based approaches that acknowledge lived histories rather than treating older adults as newly diagnoses individuals. Incorporating life-course assessment and mental health dimensions into routine HIV care illuminates how past discrimination, disclosure experiences, and social exclusion influence present-day engagement.

Psychological and psychosocial dimensions of ageing with HIV represent an important component of long-term survivorship. Psychosocial stressors may influence ageing-related health outcomes through chronic stress responses, inflammatory activation, and reduced engagement in protective health behaviors. However, much of the evidence linking psychosocial adversity and biological ageing remains associative rather than definitively causal, particularly within HIV populations. Loneliness and social isolation are discussed in greater detail below as important psychosocial determinants of survivorship.

The findings support greater integration of mental health screening and psychosocial support within HIV care systems. However, recommendations for integrated mental health services should be interpreted within the context of limited ageing-specific Philippine evidence and ongoing shortages in mental health workforce capacity.

### Depression and anxiety

Mental health integration constitutes a central pillar of survivorship reform and the expanded OPHELIA implementation. Fragmented services and shortages of trained professionals limit comprehensive psychosocial support for OPLWH (45). Depression, trauma, loneliness, and intersectional stigma undermine ART adherence and quality of life (70). Embedding mental health screening and counseling within HIV clinics, subsidizing services, and pairing digital stigma-reduction campaigns with offline peer navigation programs can create continuous and accessible support pathways. Low-cost digital initiatives expand reach, underscoring that digital efforts must be complemented by interpersonal engagement to sustain meaningful outcomes.

### Loneliness and social isolation

Psychosocial vulnerability among OPLWH may further be amplified by loneliness and social isolation. Older adults living with HIV experience shrinking social networks due to bereavement, stigma, migration, or withdrawal from previously supportive communities (12). Among older MSM and other marginalized populations, social exclusion is compounded by age-related discrimination within social and sexual networks that frequently privilege youthfulness (21). Loneliness is increasingly recognized as an important determinant of physical and mental health among PLWH and has been associated with poorer self-management, reduced care engagement, and adverse psychosocial outcomes. Emerging evidence also suggests potential links with inflammatory processes, although these relationships remain under investigation (71).

For older adults, stigma intersects with age-related invisibility in ways that may further complicate social isolation. Ageism does not operate independently from HIV-related discrimination, but rather intersects with assumptions regarding sexuality, morality, productivity, and ageing itself (21). OPLWH experience what has been described as “double invisibility,” whereby they encounter marginalization both within broader society due to age and within HIV or LGBTQ+ communities that frequently prioritize younger populations and youth-centered narratives (21). Social assumptions that older adults are asexual or no longer sexually active may contribute to diagnostic invisibility and under-screening among ageing populations (32). Consequently, HIV symptoms in older adults overlooked or attributed to normative ageing processes rather than prompting HIV testing or early intervention.

The intersecting stigmas carry psychosocial consequences. Older adults diagnosed later in life may experience profound shame or self-blame, particularly within social contexts where HIV remains associated with immorality or perceived personal failure. The lived experiences continue to shape trust in health systems, willingness to disclose HIV status, and long-term engagement in care even after viral suppression is achieved.

### Resilience and coping

Resilience remains an important and recurring dimension of HIV survivorship within the Philippines. Many PLWH describe strong support from peers, significant others, advocacy networks, and community-based organizations as essential sources of emotional stability and empowerment (72). Community-based organizations such as Gabay sa Pulang Laso Inc. provide integrated psychosocial, housing, educational, and linkage-to-care support that extends beyond biomedical treatment alone (73). For some individuals, spirituality, activism, treatment adherence, and HIV advocacy become mechanisms through which stigma is transformed into resilience, identity reconstruction, and community engagement (20). These findings underscore the importance of strength-based and culturally grounded survivorship models rather than deficit-oriented approaches alone.

Psychosocial resilience plays a role in shaping long-term outcomes for OPLWH, who may experience hopelessness contributed to declining mental health, suicidal ideation, emotional dissonance, and difficulty embracing their identity following diagnosis (72). Strong support from significant others and loved ones can help individuals confront discrimination, financial strain, and social marginalization. Individuals who maintain daily routines, prioritize nutrition, and commit to treatment adherence often demonstrate greater resilience and stability in care (72). Conversely, low social support increased psychosocial stress and the risk of psychiatric illness. A need emergers for tailored interventions for older adults, including medication optimization strategies, targeted medication reviews, adherence support, and integrated physical and psychological self-management approaches to promote sustained well-being and continuity of care (72).

### Internalized stigma

Stigma remains one of the most persistent barriers shaping HIV prevention, diagnosis, treatment engagement, and long-term survivorship in the Philippines. Although advances in ART have transformed HIV into a manageable chronic condition for many PLWH, HIV-related stigma continues to influence disclosure, mental health, social participation, and continuity of care (13, 20). For OPLWH, these experiences often compound with ageism, sexual minority stigma, poverty, and broader structural inequities, creating layered forms of marginalization that extend beyond biomedical management alone.

The psychosocial and structural dimensions of stigma also intersect closely with HL and continuity of care. Fear of disclosure, distrust of providers, prior discriminatory experiences, and concerns regarding confidentiality may discourage testing, ART initiation, or sustained clinic engagement (40). Older adults may additionally encounter challenges navigating increasingly complex systems involving multimorbidity management, specialist referrals, insurance processes, and digital health platforms. Consequently, stigma reduction strategies must extend beyond public awareness campaigns and incorporate system-level reforms that strengthen accessibility, confidentiality, cultural responsiveness, and age-inclusive communication.

Within the expanded OPHELIA framework, stigma is conceptualized not as an isolated psychosocial phenomenon but as a multidimensional determinant operating across ecological levels. Effective interventions include culturally grounded and age-inclusive HIV education, provider training in stigma-sensitive communication, peer navigation programs, community co-designed survivorship initiatives, mental health integration, and partnerships with local religious and community leaders to improve trust and reduce exclusion. Public health messaging that normalizes sexuality, ageing, HIV testing, and U=U messaging across the life course can help reduce diagnostic invisibility and internalized stigma among older adults. Addressing stigma among OPLWH in the Philippines ultimately requires coordinated approaches that integrate psychosocial support, structural equity, and health system responsiveness within broader public health planning.

## Social and structural dimensions

An underexamined dimension of ageing with HIV in the Philippines is the persistent pattern of late diagnosis among older adults. Several factors contribute to this phenomenon, including reduced perception of HIV risk among both patients and providers, sexual invisibility associated with ageing, and limited age-targeted screening initiatives.

### HIV-related stigma and ageism

In the Philippine context, HIV testing campaigns and prevention messaging have historically centered on younger key populations, particularly MSM (31). While epidemiologically justified, this emphasis may inadvertently reinforce the perception that HIV is a disease of youth.

Older adults, including heterosexual men and women, widowed individuals, and those re-entering intimate relationships later in life, may not perceive themselves as candidates for routine testing. Simultaneously, providers may not routinely offer HIV screening to older adults presenting with non-specific symptoms, attributing fatigue, weight loss, or cognitive changes to ageing rather than potential HIV infection. The consequence is diagnostic invisibility. Late diagnosis increases the duration of untreated viremia and may contribute to cumulative immune activation, potentially influencing ageing-related health outcomes over time. From a stigma perspective, delayed identification can also intensify internalized shame, as individuals may interpret diagnosis in later life as a moral or personal failure rather than a medical condition. Addressing this invisibility requires age-inclusive screening policies, provider training to reduce diagnostic bias, and communication strategies that normalize sexuality and HIV testing across the lifespan.

### Diagnostic invisibility, age-disaggregated surveillance, and provider preparedness

An underrecognized challenge within the ageing HIV epidemic is the persistent diagnostic invisibility experienced by older adults. Although HIV prevention and testing initiatives in the Philippines have appropriately emphasized younger key populations, particularly MSM, this focus may inadvertently reinforce the perception that HIV is primarily a disease of youth (31). Consequently, older adults are often excluded from prevention messaging, risk communication, and routine HIV screening efforts despite ongoing sexual activity and potential exposure risk. These dynamics may contribute to delayed diagnosis, advanced disease presentation, and prolonged periods of untreated viremia among older populations.

International evidence consistently demonstrates that adults aged 50 years and older are more likely to receive HIV diagnoses at later stages of infection, frequently presenting with lower CD4 counts and higher prevalence of AIDS-defining illnesses compared with younger cohorts (4, 74). Several interconnected factors may contribute to this phenomenon. Older adults themselves may underestimate personal HIV risk due to social assumptions surrounding ageing and sexuality, while providers similarly overlook HIV as a diagnostic consideration when evaluating older patients presenting with fatigue, weight loss, recurrent infections, or neurocognitive changes. Symptoms associated with HIV infection are mischaracterized as normative ageing processes or chronic disease rather than prompting HIV screening or referral.

These patterns reflect broader forms of age-related invisibility embedded within health systems and public discourse. Societal assumptions that older adults are sexually inactive or outside HIV “risk groups” discourages open discussions of sexual health in later life and limits opportunities for routine screening (32). In the Philippine context, where discussions surrounding sexuality are already shaped by conservative cultural and religious norms, these assumptions become further amplified among ageing populations. Older women, widowed individuals, heterosexual men, and those re-entering intimate relationships later in life remain absent from targeted HIV prevention strategies despite ongoing vulnerability.

Delayed diagnosis among older adults carries important implications for both individual survivorship and broader health system preparedness. Prolonged untreated infection contributes to cumulative immune activation, increased multimorbidity burden, and greater risk of accelerated or accentuated ageing-related complications. Late presentation additionally complicates long-term management due to concurrent chronic illnesses, polypharmacy, neurocognitive vulnerability, and delayed ART initiation (75). From a psychosocial perspective, diagnosis later in life intensifies internalized stigma or shame, particularly where HIV continues to be associated with moralized narratives surrounding sexuality and personal behavior.

The diagnostic invisibility challenges emphasize the importance of strengthening age-disaggregated HIV surveillance systems within the Philippines. Current surveillance frameworks frequently aggregate older adults into broad age categories, limiting the ability to identify emerging trends involving viral suppression, retention in care, multimorbidity burden, neurocognitive decline, frailty, and survivorship outcomes among OPLWH (34). More granular age-stratified surveillance allows earlier recognition of ageing-related service demands and strengthen workforce planning, chronic disease forecasting, and allocation of survivorship resources. Incorporating geriatric indicators into HIV monitoring systems, including frailty assessments, cognitive screening outcomes, and multimorbidity clustering, can improve preparedness for long-term HIV care in ageing populations.

At the same time, surveillance reform alone is insufficient without corresponding improvements in provider awareness and clinical preparedness. Expanding provider training represents a complementary and equally important priority. Clinicians working within primary care, HIV services, and geriatric settings require additional training regarding HIV risk and survivorship in later life, age-inclusive sexual history taking, stigma-sensitive communication, and recognition of atypical HIV presentations among older adults. Increasing provider awareness can improve earlier testing, reduce diagnostic delays, and strengthen continuity of care for ageing PLWH.

Within the expanded OPHELIA framework, surveillance reform and provider preparedness are conceptualized as interconnected components of an age-inclusive HIV response rather than separate intervention domains. Improved surveillance systems identify emerging demographic and survivorship trends, while provider education strengthens the capacity of health systems to respond effectively to those evolving needs. Together, these approaches help reduce diagnostic invisibility, improve earlier engagement in care, and strengthen long-term survivorship outcomes among older adults living with HIV in the Philippines.

### Health literacy and eHealth literacy

As the Philippines prepares for a growing population of OPLWH, survivorship outcomes will depend not only on clinical capacity but also on HL and digital inclusion. Biological ageing, multimorbidity, and integrated care models require older adults to navigate increasingly complex information environments. Reframing HIV survivorship through a HL lens positions long-term care as a dynamic interaction between individual capabilities and system responsiveness.

HL represents a foundational determinant of effective HIV management and survivorship, encompassing the cognitive and social skills that influence an individual’s ability to access, understand, evaluate, and apply health information for disease prevention and ongoing care (23, 76). Within the OPHELIA framework, HL is not viewed solely as an individual deficit but as a reflection of how well health systems align with population strengths, limitations, and contextual realities.

In contrast, eHealth literacy refers more specifically to the ability to seek, interpret, and apply health information using digital technologies, including telehealth platforms, social media, smartphone applications, and online health systems. While related to broader HL, eHealth literacy introduces additional barriers involving technological familiarity, internet access, digital navigation skills, and evaluation of online information credibility.

For OPLWH in the Philippines, several HL domains are particularly important, including medication comprehension, ART adherence navigation, understanding U=U messaging, multimorbidity management, insurance navigation, appointment coordination, and disaster preparedness planning. eHealth literacy further influences whether older adults can effectively engage with telemedicine systems, mobile adherence reminders, digital appointment scheduling, and online peer-support networks.

Despite its importance, substantial HL disparities persist nationwide. Regional inequalities, uneven access to care, hearing and sensory limitations, cognitive decline, and fragmented referral systems all reduce effective engagement among older adults. At the same time, digital expansion in the Philippines remains uneven. Urban regions demonstrate substantially greater connectivity and digital infrastructure than geographically isolated and rural communities, potentially limiting equitable implementation of telehealth-based HIV interventions.

Digital interventions nevertheless offer emerging opportunities to strengthen survivorship. Structured SMS adherence reminders, telehealth follow-up systems, and online peer support groups improve continuity of care, particularly where transportation barriers and stigma limit in-person engagement. However, digital approaches should complement rather than replace community-based and interpersonal models of care, particularly among older adults with limited digital familiarity.

Within the expanded OPHELIA framework, strengthening HL therefore requires both individual empowerment and system-level responsiveness. Age-sensitive communication strategies, simplified care pathways, culturally grounded messaging, digital inclusion initiatives, and trusted community partnerships are essential for sustaining equitable HIV survivorship in the Philippines.

### Structural fragmentation

National education policy plays a role in shaping HIV prevention efforts in the Philippines. In 2012, Congress enacted legislation to integrate reproductive health topics into formal education systems (77) and community driven or culturally grounded initiatives that have expanded the reach of HIV prevention messaging. Efforts to expand prevention in the Philippines incorporates civil society organizations, artists, faith groups, schools, and local advocates that collaborate to address gaps in HIV knowledge and increase community engagement (78). Through digital media and multimedia platforms, community-based organizations and artist produced prevention campaigns frame HIV within broader themes of identity, social connectedness, and collective responsibility (31, 79). The cultural approach emphasizes community co-design, responsiveness to local literacy strengths and limitations, and tailoring interventions to reflect lived experiences. By embedding prevention messaging within culturally resonant narratives, the initiatives help normalize conversations about HIV and counter stigma in a manner that traditional biomedical messaging along may not achieve (78, 80).

As of 2022, the Philippines had not implemented a comprehensive national policy and health care access remained a critical barrier (43). Although ART is provided for free by the Philippine Department of Health, structural limitations, including uneven geographic distribution of services, practitioner shortages, and fragmented care delivery, suppress effective HL by increasing system complexity (3). Regional disparities are evident: the National Capital Region reports higher HL (65.4%) compared with Luzon (48.2%) and Mindanao (49.2%) (23). Older adults demonstrate the highest levels of limited HL, influenced by hearing loss, reduced mobility, declining reasoning abilities, and sensory impairment (22). For OPLWH, age-related cognitive changes may intersect with HIV-associated neurocognitive vulnerability, yet Philippine research has not sufficiently disaggregated data to examine this subgroup. From an OPHELIA perspective, this represents a critical evidence gap requiring age-sensitive data assessment to inform tailored survivorship interventions.

Public trust remains a critical determinant of national policy effectiveness. Vaccine hesitancy in the Philippines, with 41% of surveyed individuals expressing refusal of the COVID-19 vaccine, which reflects fragile confidence in public health institutions (81). The lingering impact of the dengue (Dengvaxia) vaccine controversy underscores how erosion of trust can disrupt uptake of health interventions (24). These dynamics have implications for HIV prevention, U=U communication, and potential future biomedical innovations. Within OPHELIA, strengthening HL requires transparent communication, gender-sensitive outreach, and sustained community engagement to rebuild confidence in health systems (76). For older adults who may rely heavily on interpersonal trust and longstanding community networks, restoring credibility is central to sustained survivorship engagement.

Despite progress in community outreach efforts, persistent knowledge gaps continue to influence prevention behaviors, particularly regarding biomedical interventions such as PrEP. Misconceptions about PrEP protocols, concerns about side effects, and lingering beliefs that an HIV diagnosis constitutes a death sentence remain barriers to broader uptake (50, 70, 82). In Davao City, disparities in awareness, attitudes, and willingness to use PrEP emphasize uneven diffusion of prevention knowledge (83). Filipinos commonly report obtaining HIV and PrEP information through social media, peer networks, and government clinics, suggesting variability in the accessibility, clarity, and trustworthiness of available information (45, 84). Awareness is highest among members of the gay community and lowest among heterosexual populations, reflecting uneven targeting of health communication strategies (83).

The disparities, signal not only informational deficits but also mismatches between community fragmentation and the design of prevention services. Individuals who lack PhilHealth coverage, government-issued identification, or a permanent address often cannot access laboratory services promptly and must delay receiving test results and medications (58). In this context, strengthening prevention efforts requires more than expanding information dissemination, rather it necessitates tailoring communication strategies, simplifying service navigation pathways, and embedding PrEP education within trusted community structures (85). Closing knowledge gaps through targeted outreach, inclusive messaging, and system-level adaptations will be essential to maximizing the preventive potential of PrEP nationwide.

### Workforce capacity, digital infrastructure, and age-responsive HIV preparedness

As the population of OPLWH continues to expand in the Philippines, the long-term effectiveness of survivorship-oriented HIV care will depend not only on biomedical management, but also on the capacity of health systems to respond to increasingly complex and age-responsive care needs. Several structural issues identified throughout this review, including workforce shortages, fragmented service delivery, limited age-disaggregated surveillance, and uneven digital infrastructure, highlight important preparedness gaps that influence continuity of care for ageing PLWH in the coming decades.

One critical challenge involves the growing need for workforce development integrating both HIV-specific and geriatric competencies. As a collective, HIV clinicians have limited formal training in frailty assessment, multimorbidity management, neurocognitive screening, and age-related pharmacologic considerations, where as geriatric providers possess limited experience managing ART regimens, HIV-associated neurocognitive disorders, or HIV-related stigma (8, 19). As survivorship improves and multimorbidity becomes increasingly prevalent among OPLWH, these competency gaps contribute to fragmented care pathways and reduced continuity of long-term management.

The urgency of workforce strengthening is further amplified by broader structural pressures within the Philippine health system. Geographic fragmentation across more than 7,600 islands, uneven distribution of health facilities, and substantial migration of Filipino health professionals abroad continue to constrain service availability and continuity (18). These challenges are significant for older adults requiring multidisciplinary management involving HIV care, chronic disease treatment, mental health services, cognitive screening, and social support systems. Although direct national data examining HIV-geriatric workforce preparedness remain limited, existing evidence regarding clinician shortages and decentralized governance suggests that integrated competency development will become increasingly important as the HIV population ages.

Within the context of ageing with HIV, workforce development extends beyond biomedical expertise alone. Providers require additional training in stigma-sensitive communication, age-inclusive HIV screening practices, mental health assessment, digital health navigation, and culturally responsive survivorship counseling. Diagnostic invisibility among older adults remains a persistent concern internationally and occurs within the Philippines, where HIV prevention campaigns have historically emphasized younger MSM populations (31). Older adults presenting with fatigue, weight loss, neurocognitive changes, or recurrent infections can experience delayed HIV testing if symptoms are attributed primarily to normative ageing rather than potential HIV infection (74). Expanding provider awareness regarding sexuality, HIV risk, and survivorship in later life strengthens earlier diagnosis and reduce age-related disparities in care engagement.

Digital infrastructure and technological inclusion represent another emerging dimension of age-responsive HIV survivorship. Telehealth systems, SMS adherence reminders, online peer-support groups, and mobile health interventions have demonstrated potential to strengthen treatment adherence, continuity of care, and psychosocial engagement among PLWH (86, 87). Programs such as Connect for Life Philippines have shown improvements in ART adherence through structured communication systems integrating reminders, symptom monitoring, and appointment support (86). These approaches hold particular relevance within geographically fragmented settings where transportation barriers, stigma, and provider shortages limit in-person engagement.

However, digital expansion within the Philippines remains uneven. Internet connectivity, smartphone access, and digital literacy vary substantially across urban and rural regions, potentially limiting equitable implementation of telehealth-dependent HIV services (88). Older adults may encounter additional barriers related to financial limitations, reduced technological familiarity, sensory decline, or difficulty evaluating online information credibility (22). Consequently, digital interventions should not be conceptualized as universal replacements for interpersonal or community-based care systems. Rather, telehealth and digital tools function most effectively when integrated alongside peer navigation, community outreach, and decentralized service models that remain accessible to populations with limited technological resources.

The intersection of ageing and digital migration with HIV care further complicates survivorship. Health communication in the Philippines increasingly occurs through digital platforms, telehealth systems, and social media campaigns (89). While digital tools enhance access to medication reminders, appointment scheduling, and peer support, digital literacy varies widely among older adults (87). Barriers include language limitations, financial constraints, limited baseline knowledge, and difficulty navigating online platforms (88). For OPLWH, the shift toward digital engagement either empowers self-management or exacerbates exclusion.

Media and popular culture also shape HIV-related HL through networks of participation and influence. Filipino television dramas and entertainment platforms portrayed the lived experiences of people with HIV, expanding public discourse and challenging stigma (86). The media portrayals contribute to normalization and empathy in a sociocultural context where discussions of sexuality encounter resistance (50). For older adults, whose attitudes are shaped by earlier eras of intense stigma, such media narratives can recalibrate understanding (80, 90) and support U=U awareness. Promoting U=U among older adults is important, as misconceptions about transmission risk perpetuate internalized stigma and social withdrawal. Embedding U=U messaging within culturally resonant media and community channels aligns with OPHELIA’s emphasis on system-level communication strategies that build trust and comprehension.

The findings collectively highlight that ageing with HIV in the Philippines will require more than expansion of ART access alone. Effective survivorship planning must integrate workforce preparedness, digital inclusion, age-responsive surveillance, and culturally grounded continuity-of-care systems capable of addressing the biological, psychosocial, and structural complexity of ageing with HIV. Within the expanded OPHELIA framework, these dimensions are conceptualized as interconnected components of a literacy-responsive and equity-centered health system rather than isolated intervention targets. Strengthening preparedness for OPLWH therefore requires coordinated investment in provider training, digital accessibility, surveillance reform, and integrated survivorship infrastructure capable of adapting to the evolving demographic realities of the Philippine HIV epidemic.

### Cultural and religious influences

HIV-related stigma shapes moral, religious, and sociocultural narratives surrounding sexuality, gender, and perceived deviance in the Philippines (20, 46). In a predominantly Catholic society, discussions of sexuality often remain constrained by conservative social expectations, while some religious institutions continue to frame same-sex behavior and HIV within moralized or sinful frameworks (13). Although the Philippines is frequently regarded as comparatively accepting of LGBTQ+ populations within Southeast Asia, structural inequities persist through the absence of legal recognition for same-sex unions, limitations in family rights, and continuing heteronormative social expectations (20). These dynamics contribute to delayed testing, nondisclosure, internalized stigma, and disengagement from formal health systems among PLWH.

Culturally responsive care remains central to effective HIV survivorship. Among the Maranao population, health beliefs are strongly shaped by family, community, dignity, and Islamic faith (46). Preferences for traditional healing and religious practices influence health-seeking behavior. HIV related stigma within some communities reinforces social exclusion and delays engagement in care. Such integrated models demonstrate that survivorship extends beyond viral suppression to encompass housing stability, employment, social inclusion, and mental well-being. Culturally appropriate programs exemplify health system responsiveness to complex literacy and access needs. Addressing structural vulnerabilities alongside clinical care sustains engagement among OPLWH who may otherwise face compounded risks of poverty, stigma, cognitive decline, and isolation.

## Translational and health systems response

OPHELIA emphasizes co-designing interventions with communities to align services with cultural HL profiles. For OPLWH, survivorship programs must collaborate with religious and community leaders to integrate HIV education, chronic disease management, and cognitive health awareness within culturally congruent frameworks (46). Failure to align services with cultural values risks disengagement, particularly among older populations with deeply rooted belief systems.

### OPHELIA implementation pathways

The OPHELIA framework provides a structured pathway for translating multidimensional evidence into actionable and equity-centered public health interventions. While the original framework emphasizes situational assessment, co-design, implementation, and evaluation responsive to population HL needs, the present manuscript expands the framework to address the emerging realities of ageing with HIV in the Philippines. The expanded framework incorporates four interconnected implementation domains: age-responsive survivorship assessment, integrated HIV-geriatric service delivery, health literacy and digital inclusion, and climate-resilient continuity planning.

Within the Philippine context, implementation may include routine frailty and neurocognitive screening within HIV clinics, age-inclusive HIV testing campaigns, telehealth-supported adherence systems, peer navigation programs, and culturally grounded stigma-reduction interventions developed in partnership with community organizations and faith leaders.

The framework also emphasizes workforce development. HIV clinicians require greater geriatric competencies related to multimorbidity management, frailty assessment, polypharmacy review, neurocognitive screening, and stigma-sensitive communication. Conversely, geriatric providers benefit from additional HIV-specific training related to ART management, HIV survivorship, and intersectional stigma.

Importantly, implementation should remain responsive to geographic fragmentation and unequal digital infrastructure across the Philippines. Telehealth systems, SMS adherence interventions, and online support networks may strengthen continuity of care, but these approaches should complement rather than replace in-person and community-based care pathways.

The expanded OPHELIA approach therefore functions not only as a HL model but also as a broader systems-level framework for integrating ageing, survivorship, stigma reduction, and equity-centered implementation within HIV care.

### Workforce development

The Philippine health system operates across provincial, city or municipal, and barangay levels. As of 2018, the country had 1,198 hospitals and 2,593 primary care facilities, yet only about half of the population lives within a 30-min radius of a health facility (18). Workforce shortages further constrain care delivery, with approximately one in five Filipino health professionals working abroad (18).

Many HIV clinicians have limited formal training in gerontology, while geriatricians may have insufficient exposure to HIV specific management (19). Addressing survivorship requires cross-training initiatives that build competencies in multimorbidity management, age-related pharmacokinetics, cognitive screening, and stigma sensitive communication (8). Volunteers often bridge service gaps, yet many work long hours without compensation, reflecting ongoing strains within the health system. Despite the barriers, many individuals and community advocates integrate HIV advocacy with personal resilience, transforming lived experience into pathways for healing, growth, and renewed purpose (58).

These structural constraints highlight mismatches between system complexity and workforce capacities. The expanded OPHELIA framework provides a way for OPLWH to navigate ART adherence, insurance claims, dementia screening, and specialty referrals to interpret and act upon complex health information. Optimizing survivorship outcomes therefore requires simplifying care pathways, improving system navigation support, and strengthening primary care integration at the community level.

### Integrated care models

Strengthening HIV survivorship for older adults in the Philippines requires system-level reform that integrates financing, workforce development, digital infrastructure, culturally grounded prevention, and mental health services within a HL–responsive framework. The Philippine Universal Health Care Act 11223 (UHC) establishes an important structural foundation by promoting intermunicipal cooperation, strengthening primary care networks, expanding workforce capacity, and leveraging digital health technologies to improve coordination and information exchange (4, 89). For OPLWH, effective UHC implementation must reduce fragmentation across administrative boundaries and simplify navigation of long-term, multidisciplinary care (43). Strengthening provider confidence and competence is particularly important in addressing intersectional stigma, including HIV related discrimination and ageism, which shape care experiences and trust in health systems (24). Integrated governance structures, trust-based partnerships between national and local agencies, and transparent monitoring systems are essential to ensuring equitable survivorship access across regions.

Prevention policy reform also remains critical for long-term survivorship. As of 2022, the Philippines lacked a formal national regulatory framework for HIVST (43). Although often framed as a prevention tool, HIVST also influences survivorship trajectories by facilitating earlier diagnosis and timely ART initiation, thereby mitigating long-term biological ageing effects (91). Policymakers should proactively regulate pricing, distribution, and quality assurance of HIVST kits while integrating self-testing into broader continuity-of-care pathways (42, 43). Embedding HIVST within community-based and digitally supported models aligns with reducing access barriers and tailoring interventions to diverse literacy and geographic contexts.

Collectively, aligning UHC reforms, digital workforce training, HIVST regulation, culturally grounded harm reduction, and integrated mental health services creates a coherent survivorship strategy. Rather than approaching these domains as isolated policy initiatives, this synthesis positions the domains as interconnected components of a responsive system designed to optimize HL and access for OPLWH. By embedding survivorship within national reform agendas and grounding implementation in community co-design, the Philippines can strengthen resilience, reduce disparities, and ensure that ageing with HIV is supported by coordinated, equitable, and person-centered care.

### Climate resilience and continuity planning

HL also intersects with disaster preparedness in a country highly vulnerable to typhoons, flooding, and other natural hazards. The Philippines experiences an average of 20 typhoons annually, and climate related disasters disrupt clinics, interrupt ART supply chains, delay patient enrollment, and heighten psychological stress (47, 59). Disaster response disruptions interrupt ART access, displace older adults, and fracture social support networks (59). The importance of emergency preparedness plans emerges to include clear, accessible communication about medication continuity, mobile clinic deployment, food security, and telehealth alternatives (16, 47, 76, 88, 92). Beyond individual literacy to system resilience, supporting decentralized medication distribution, telehealth counseling, mobile clinics, and disaster integrated ART planning. Building climate-resilient, literacy responsive infrastructure is central to sustaining viral suppression and ensuring preparedness for an ageing population living with HIV in the Philippines.

## Discussion

The Philippine HIV epidemic is entering a critical transitional phase. While incidence continues to rise, particularly among MSM, expanding ART access is simultaneously increasing survivorship. These parallel dynamics position the Philippines at an inflection point where a rapidly growing epidemic will increasingly confront the clinical, psychosocial, and structural demands of an ageing population living with HIV. Unlike countries such as Thailand, where ageing cohorts reflect longstanding epidemic control, the Philippines faces a dual burden of ongoing transmission and increasing longevity. Consequently, ageing-related morbidity is likely to accumulate within a health system already challenged by workforce shortages, decentralized governance, and geographic fragmentation. Current gaps in testing, ART initiation, and sustained viral suppression further increase the likelihood that individuals will enter older age with prolonged exposure to uncontrolled viremia and chronic immune activation.

A recurring limitation in the Philippine HIV response is the lack of age-disaggregated surveillance. Without systematic monitoring of multimorbidity, frailty, neurocognitive outcomes, and mental health indicators across older age cohorts, the magnitude of ageing-related HIV burden may remain underestimated (34). More granular surveillance of adults aged 50–59, 60–69, and 70 years and older could improve workforce planning, resource allocation, and early identification of emerging survivorship needs. Incorporating geriatric indicators, including frailty indices, validated cognitive screening tools, and multimorbidity measures, would further support proactive care planning and institutional recognition of older adults as a distinct and growing population within the epidemic.

The ecological framework of accelerated and accentuated ageing provides a biologically plausible explanation for the earlier onset and increased prevalence of age-associated comorbidities among PLWH. Chronic immune activation, immunosenescence, and persistent inflammation (“inflammaging”) have been associated with cardiovascular disease, neurocognitive impairment, osteoporosis, and metabolic disorders (93, 94). Although the precise biological pathways remain under investigation, multimorbidity and polypharmacy are likely to become increasingly common as cohorts age, reinforcing the need to integrate geriatric principles within HIV care. Frailty screening, medication review, and routine cognitive assessment should become standard components of longitudinal survivorship services. Importantly, biological ageing does not occur in isolation. Stigma, depression, loneliness, and social exclusion have been associated with reduced care engagement and adverse health outcomes, suggesting that biomedical and psychosocial interventions should be viewed as complementary rather than discrete domains.

The sociocultural context of the Philippines further shapes ageing with HIV. Religious norms, moral framing of sexuality, criminalization of certain behaviors, and age-related marginalization intersect to produce layered forms of stigma. Older adults may experience invisibility within prevention messaging, exclusion from social networks, and internalized ageism that discourages engagement in care. Qualitative evidence reviewed in this paper suggests that loneliness and social isolation may be particularly salient among older MSM, with implications for mental health, adherence, and quality of life.

Health literacy remains a critical determinant of survivorship. Older adults demonstrate lower average HL levels nationally, while digital literacy gaps may limit engagement with telehealth and mHealth interventions. Strengthening HL therefore requires more than prevention education; it must encompass multimorbidity management, medication literacy, health system navigation, and disaster preparedness. Age-sensitive communication strategies, promotion of U=U messaging, faith-sensitive outreach, and community-based peer support may help improve comprehension, engagement, and continuity of care. Digital interventions—including SMS reminders, telehealth systems, mobile applications, and online peer-support platforms—offer promising tools to support adherence and navigation but should complement rather than replace interpersonal and community-based models of care.

As multimorbidity, frailty, neurocognitive decline, and polypharmacy become increasingly prevalent among OPLWH, workforce preparedness will become equally important. HIV clinicians may require greater competencies in frailty assessment, neurocognitive screening, medication management, mental health assessment, and care coordination, while provider awareness regarding sexuality and HIV risk in later life may help reduce delayed diagnosis and age-based assumptions about risk. Expanding competencies across both HIV and geriatric care settings may reduce fragmentation, improve continuity of care, and strengthen age-responsive service delivery.

Structural determinants, including workforce migration, uneven facility distribution, and climate-related disasters, present additional challenges to continuity of care. The Philippines’ vulnerability to typhoons and flooding creates recurring risks to ART supply chains, clinic access, and social support systems. For older adults with mobility limitations and multimorbidity, these disruptions may have amplified consequences. Integrating climate resilience into HIV programming through decentralized medication distribution, emergency refill protocols, telehealth support, and disaster preparedness planning should therefore be considered an essential component of ageing-responsive care.

To translate these multidimensional findings into actionable policy, this paper advances an expanded application of the OPHELIA framework. Originally designed to strengthen HL through community co-design and iterative implementation, OPHELIA provides an equity-centered model adaptable to ageing with HIV. The expanded framework incorporates age-sensitive situational assessment, including multimorbidity mapping, frailty screening, stigma archetype identification, and digital capacity evaluation; co-design of geriatric-integrated and stigma-responsive interventions with OPLWH, clinicians, faith leaders, and community organizations; and broader evaluation metrics that extend beyond viral suppression to include functional status, mental health, HL, and resilience to disaster-related disruptions. By linking biological ageing mechanisms with community-driven HL strategies, the adapted OPHELIA framework bridges mechanistic science and public health implementation while offering a scalable model for ageing HIV populations in the Philippines and other rapidly evolving epidemics in the Asia-Pacific region.

## Conclusion

Several research priorities emerge. First, biomarker validation studies within Filipino cohorts are needed to determine whether patterns of epigenetic age acceleration and immunosenescence observed in high-income settings are replicated locally. Second, implementation science studies should evaluate the feasibility and cost-effectiveness of integrating frailty screening and mental health services within HIV clinics. Third, longitudinal surveillance should track ageing-related outcomes, including cognitive impairment and polypharmacy burden. Policy reforms should prioritize integration of HIV and geriatric competencies within workforce training, strengthen HL initiatives targeting older adults, and embed climate resilience into service planning. Financing mechanisms must anticipate increased demand for chronic disease management and social support services among ageing cohorts.

This analysis synthesizes existing epidemiologic and theoretical literature and therefore depends on the quality and availability of current data. Age-disaggregated data specific to OPLWH in the Philippines remain limited, underscoring the need for improved surveillance and primary research. Additionally, while theoretical mechanisms of accelerated ageing are well supported in global literature, direct validation in Filipino populations is needed.

Finally, future policy and research must more explicitly center the voices of OPLWH. Participatory research methodologies, community advisory boards inclusive of older members, and qualitative inquiry into age-specific stigma experiences can strengthen the cultural validity of reform efforts. Without direct engagement, policies risk perpetuating assumptions about older adults’ needs rather than responding to articulated priorities. Empowering older Filipinos living with HIV to contribute to service design aligns with equity-centered public health principles and strengthens sustainability.

The Philippines stands at a pivotal juncture in its HIV response. As survivorship improves, ageing-related morbidity will increasingly shape the epidemic’s trajectory. Addressing this emerging burden requires integration of biological ageing science, psychosocial insight, structural resilience, and equity-centered implementation frameworks. The expanded OPHELIA approach offers a coherent strategy for translating these multidimensional insights into age-responsive action. Proactive investment in surveillance, integrated care models, HL strengthening, and stigma reduction can position the Philippines to mitigate the long-term consequences of ageing with HIV while advancing sustainable epidemic control.
